# Ageing of Dental Composites Based on Methacrylate Resins—A Critical Review of the Causes and Method of Assessment

**DOI:** 10.3390/polym12040882

**Published:** 2020-04-10

**Authors:** Agata Szczesio-Wlodarczyk, Jerzy Sokolowski, Joanna Kleczewska, Kinga Bociong

**Affiliations:** 1 University Laboratory of Materials Research, Medical University of Lodz, Pomorska 251, 92-213 Lodz, Poland; kinga.bociong@umed.lodz.pl; 2 Department of General Dentistry, Medical University of Lodz, Pomorska 251, 92-213 Lodz, Poland; jerzy.sokolowski@umed.lodz.pl; 3 Arkona: Laboratory of Dental Pharmacology, Nasutów 99C, 21-025 Niemce, Poland; joanna@arkonadent.com

**Keywords:** dental resins, fillers, dental composite, ageing, degradation

## Abstract

The paper reviews the environmental factors affecting ageing processes, and the degradation of resins, filler, and the filler-matrix interface. It discusses the current methods of testing materials in vitro. A review of literature was conducted with the main sources being PubMed. ScienceDirect, Mendeley, and Google Scholar were used as other resources. Studies were selected based on relevance, with a preference given to recent research. The ageing process is an inherent element of the use of resin composites in the oral environment, which is very complex and changes dynamically. The hydrolysis of dental resins is accelerated by some substances (enzymes, acids). Bonds formed between coupling agent and inorganic filler are prone to hydrolysis. Methods for prediction of long-term behaviour are not included in composite standards. Given the very complex chemical composition of the oral environment, ageing tests based on water can only provide a limited view of the clinical performance of biomaterial. Systems that can reproduce dynamic changes in stress (thermal cycling, fatigue tests) are better able to mimic clinical conditions and could be extremely valuable in predicting dental composite clinical performance. It is essential to identify procedure to determine the ageing process of dental materials.

## 1. Introduction

Composites based on a polymer matrix are increasingly used in various industrial fields. As a consequence, they need to provide long-term service in hostile environments [1]. Despite sophisticated design methods and great care in production, irreversible changes in the original properties are inevitable and this effectively limits the operating life of the restoration. Such deterioration over time is referred to as ‘ageing’ [2].

Since their introduction to the market over 50 years ago, dental composites have been used in a wide range of applications and their popularity is continuously growing [3]. This growth has been attributed to their [3,4,5]:

- outstanding aesthetic properties
- strength and toughness, comparable to dental amalgams and porcelain (flexural, compression and tensile strength)
- ease of use and modelling.

Dental restorations are still beset by lack of consistent degree of conversion, limited fracture resistance, wear, and polymerization shrinkage stress [4]. The use of dental composites also involves the risk of micro-leakage, with secondary caries as a possible consequence. Improvements are constantly being sought to reduce the risk of treatment failure. One recent advance in dental composites is the use of nanotechnology in the production of fillers. Materials with such a modified inorganic part are characterized by high mechanical strength, high abrasion resistance, improved optical properties, and reduced polymerization shrinkage [6]. In addition, they may show increased resistance to acidic solutions [7].

All of these factors influence the lifetime of a composite restoration, and are therefore of great interest among clinicians. It was observed that premolar and molar restorations need to be replaced after five or six years [8,9]. An evaluation of approximately 100,000 clinical outcomes indicated that the probability of survival of a composite restoration for seven years is 92% [10]. However, other surveys report that small to moderate-sized composite restorations demonstrate effective long-term performance for 10 or more years [11,12]. In addition, 17- and 22- year studies indicate that posterior composite restorations display acceptable clinical performance after long-term evaluation [13,14,15]. Composite restoration are most commonly replaced due to fracture, secondary caries [9,16,17,18], and wear [11]. As the ageing process of dental composites is a key consideration in their use in the oral cavity, a thorough review of this topic is necessary.

## 2. Materials and Methods

Sources: A review of literature was conducted with the main source being PubMed. ScienceDirect, Mendeley, and Google Scholar were used as other resources. The review was followed by a manual search of citations from relevant articles. The following keywords were used during searching: degradation, ageing, aging, dental, dentistry, composite, resin, methacrylate, dimethacrylate, fillers, filler-matrix interface. The search prioritized studies published in the last twenty years, although it also includes older papers. In addition, selection of research published in the last five years was carried out. Over 11,000 records were identified. Changing the search and sorting parameters resulted in approximately 5000 records being screened. From them, 167 were selected based on relevance to this review.

Study selection: An outline of the most important aspects of the ageing process of dental composites was created. Studies were then selected based on relevance, with recent research given priority.

## 3. Oral Environment Characteristics

**Figure 1:**
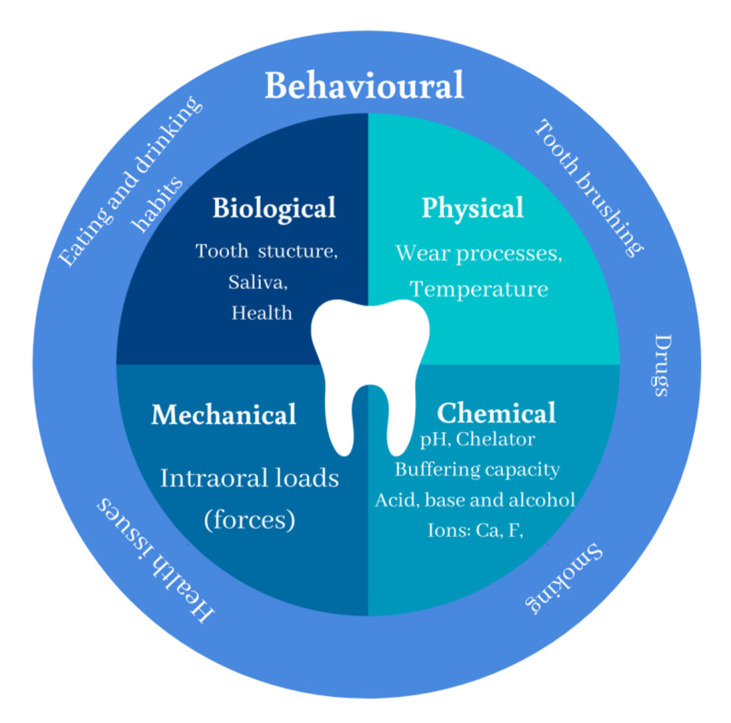
Characteristic of oral environment divided into four main groups: chemical, mechanical, biological, and physical factors. *Behavioural aspects affect individual factors.

Composite ageing is affected by chemical, physical, and mechanical processes; these may interact with each other depending on the characteristics of the material, the environment, and mechanical load [2]. Dental materials are exposed to a highly-complex and dynamically-changing environment (Figure 1). In addition, behavioural factors are individual and may have a drastic influence on the oral environment and its associated physiological processes.

### 3.1. Biological Factors

Saliva contains water, histamines, statins, lysozyme, proline-rich proteins, carbonic anhydrase, amylases, peroxidases, lactoferrin, mucins, and secretory immunoglobulin A (sIgA). These substances perform a number of functions including, buffering, digestion, lubrication, tissue coating, mineralization, antiviral, and antibacterial effects [19,20].

Bacteria form a pellicle on the surface of the tooth, covering both oral hard and soft tissues. It is a biofilm composed of mucins, glycoproteins, and proteins, including several enzymes [21]. The presence of a pellicle allows the attachment of various bacterial species, which co-aggregate, forming a mature oral biofilm [22]. Some of these bacteria produce organic acids, including lactic, acetic, formic, and propionic acids, during metabolism of carbohydrates. Although these components have been shown to readily dissolve the mineral of the enamel and dentine, saliva normally maintains the pH near neutral value (pH = 7). In addition, the flow of saliva removes acids from bacterial activity as well as other carbohydrates that could be further metabolized by bacteria. The buffering capacity of saliva also neutralizes the acidity from drinks and foods [23].

### 3.2. Chemical Factors

In addition, dental composites are exposed to various compounds (acids, alkalis, salts, alcohols, oxygen, etc.), which enter the mouth while eating and drinking. Furthermore, the total amount and frequency of consumption of acidic foods and drinks respond to changes in lifestyle; for example, in the USA, the consumption of soft drinks increased by 300% over the past 20 years and meal have also increased drastically [24,25]. Smoking tobacco and drinking alcohol increase the chance of restoration failure [26].

Although the environment in the mouth is maintained near neutrality, most food is acidic. The pH of saliva typically ranges from 6.2 to 7.6 [27,28].

### 3.3. Physical Factors

Physical factors also determine the conditions in the oral cavity. The basic aspect is the varying temperature in the mouth. The mean temperature of the mouth varies by about 36 °C, with the highest reported temperature being over 50 °C and the lowest about 5 °C [29]. Additionally, teeth are subject to wear processes such as abrasion (mechanical processes involving foreign substances or objects observed during mastication or tooth brushing), attrition (a result of the action of antagonistic teeth—no foreign substances intervening), abfraction (result of stresses in the cemento-enamel region, which may generate microfractures in enamel and dentine), and erosion (chemical wear as a result of acids or chelators acting on plaque-free tooth surfaces) [30]. Composite wear is affected by the structure and surface of the material, interaction conditions, and environmental factors [31].

### 3.4. Mechanical Factors

The transfer of external biting loads is one of the key tasks of teeth structure. Intraoral loads (forces) have been reported to range from 10 to 430 N. A functional load of 70 N is considered as clinically normal [32]. Other researchers claim that maximum bite force is over 800 N [33]. Load per tooth depends on the number of teeth, type of occlusion, and behavioural habits of patients (e.g., bruxism). Additionally, a restored tooth tends to transfer stress differently to an intact tooth. The normal tooth structure transfers loads through enamel into dentin as compression stress; however, in a restoration, any force causes occurrence of compression, tensile, or shear stresses along the tooth-restoration interface. Mechanical loads also cause material ageing [32].

“Dental composites consist of an organic part and an inorganic filler that shapes the properties of materials. Both phases are connected via a coupling agent and the physical and mechanical properties of the material depend on the composition and content of both phases” [34]. Composite materials also include additional substances, such as polymerization initiators, inhibitors, and catalysts responsible for crosslinking process, dyes, antioxidants, and UV stabilizers [35].

## 4. Degradation of Resins

The difference between degradable and non-degradable polymers is not clear-cut, because degradation and erosion are inherent processes associated with polymers. In order to differentiate the two, the timescale and usage of the material should be taken into account. If the material degrades over time or immediately after use, it is referred to as degradable; while those that will not degrade during the intended use of the material are non-degradable [36]. Polymer degradation can be defined as “a deleterious change in the chemical structure, physical properties, or appearance of a polymer, which may result from chemical cleavage of the macromolecules forming a polymeric item, regardless of the mechanism of chain cleavage” [37]. This process is caused by various mechanisms, such as photo, thermal, mechanical, and chemical processes. Degradation may change the mechanical, optical, electrical, and colour characteristics of the material and cause erosion and phase separation [38]. Therefore, for practical purposes, it is essential to determine the useful lifetime (exploitation time) of the polymer in the service environment. In most cases, polymer lifetime is estimated through the use of accelerated ageing with increased environmental factors, for example temperature, pH or radiation. This approach measures degradation rate under controlled conditions and then estimates the time that the material would reach failure point under these conditions. The rate of hydrolytic degradation depends on applied method (conditions, water diffusivity in the polymer matrix, device dimensions) and the nature of the polymer (composition, molecular weight distribution, swellability, geometry of polymer matrix, porosity) [37].

The basis of the organic phase of most contemporary dental composites is 2,2-bis-[4,4-(2′-hydroxy-3’-methacryl-iloxypropoxy)-phenyl]-propane resin (bis-GMA). The disadvantage of this monomer is the high hydrophilicity of the resin, resulting from the presence of hydroxyl groups in the molecule. Another problem is its high viscosity, which is caused by strong intermolecular interactions and the formation of hydrogen bonds between macromolecules. Such high viscosity delays the sedimentation of the filler particles and slows its homogeneous dispersion in the polymer matrix [39]. To prevent excessive density of composites and give them desirable performance, it is necessary to use bis-GMA resin in combination with low viscosity monomers, such as triethylene glycol dimethacrylate (TEGDMA), ethylene glycol dimethacrylate (EGDMA), ethylene diglycol dimethacrylate (DEGDMA), 2-hydroxyethyl methacrylate (HEMA), and 1,10-decanediol dimethacrylate (DDDMA or D3MA). The organic phase undergoes solidification via free radical chain polymerization. This process can be initiated by a photochemical reaction (the most common) or by a chemical reaction of initiator and co-initiator. Polymerization leads to the formation of a cross-linked network. The resulting polymer network is characterized by esters, urethanes, amides, hydrogen bonds, and van der Waals interactions [40].

As a result of their chemical structure, methacrylate dental resins are prone to water sorption. Water may be entrapped in material during photopolymerization or enter the polymer matrix after curing by diffusion. For polymers, two major models of diffusion have been developed. The first one—the “free volume theory”—assumes that water penetrates resin matrix through nanopores, without any chemical reaction with polymer chains. The second model—the “interaction theory”—proposes that water diffuses through the material by binding to hydrophilic groups [41]. In TEGDMA, there are ethylene oxide groups that have a high affinity for water molecules. Other monomers, such as urethane dimethacryate (UDMA) and bis-GMA, contain functional groups such as urethanes and hydroxyls, respectively, that also may bind water molecules [42]. The absorbed water occurs in two forms: unbound water, which occupies free volume and nanopores between the polymer chains created during polymerization, and bound water, which is attached to polymer chains by chemicals interactions such as van der Waals or hydrogen bonds [43].

Water is responsible for the chemical hydrolysis of ester bonds in methacrylates. This process depends on the type of bond. Ester binding is more susceptible to nucleophilic attack by water than carbonate, carbamate, urethane or amide bonds, and hence occurs more readily at physiological pH [42]. Normally hydrolysis proceeds slowly in the neutral conditions of the oral cavity, but it can be accelerated by the conditions in the surrounding environment. It may be catalyzed by acids, bases, or enzymes derived from bacterial activity, as well as food and oral physiology [44]. Five major groups of enzymes in the oral cavity have been determined: transferring enzymes, carbohydrates, esterases and proteolytic enzymes, as well as others such as carbonic anhydrase [45]. Of these, the esterases are the most extensively studied with regard to their effect on resin composites. It has been reported that esterase derivatives isolated from human saliva catalyse the hydrolytic breakdown of dental methacrylate resins [46,47,48,49,50]. Many esterases have been used to assess the degradation of resin composites and some of them now are used to investigate the stability of new resin technologies.

**Figure 2:**
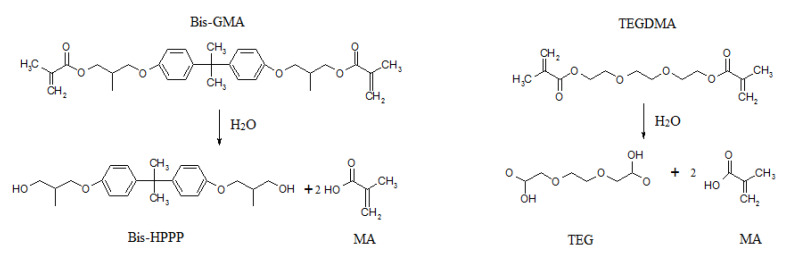
Mechanism of hydrolysis processes of BisGMA and TEGDMA.

Chemical degradation of resin results in the release of degradation products, and the deterioration of the structure and mechanical properties of the material. The main final product of the hydrolysis of methacrylates is methacrylic acid (MA). The degradation of Bis-GMA and TEGDMA results in the formation of bishydroxy-propoxy-phenyl-propane (BisHPPP) and triethylene glycol methacrylate (TEGMA). Further breakdown of TEGMA leads to the creation of triethylene glycol (TEG) (Figure 2) [46,51,52]. Progressive degradation results in further swelling polymeric matrix with water sorption, which allows for unreacted monomers and degradation products to diffuse out of the composite more easily. The degree of monomer conversion (DC) is a very important factor for the stability of the composite material. DC can amount to 55–85% [53]; however, some researchers claim that only 60% of the total number of monomer molecules are bound in the polymerized composite [40,54].

Unbound monomers and degradation products diffuse through the dentinal tubules into the pulp and other surrounding tissues and finally into the bloodstream. The biological risk of using dimethacrylate resins includes acting as potential carcinogens, immunological reactions, and estrogenicity [55]. Additionally, greater material susceptibility to abrasive wear is observed during mastication, as a result of water sorption and hydrolytic degradation of the polymer. This phenomenon could be explained by surface softening [56,57]. With gradual abrasion, subsequent layers of material are exposed to chemical degradation.

As hydrolytic degradation can be catalyzed by substances found in the changing oral environment, it is important to create a resistant polymer matrix.

## 5. Degradation of Filler and Filler-Matrix Interface

In order to improve physical properties of polymers, fillers are introduced into their composition. Fillers are used in dental composites to increase strength, refractive index, and change the thermal expansion coefficient of the material [58,59,60]. An increase in filler content should reduce the shrinkage of the material following polymerization and the resulting shrinkage stress by reducing the content of the organic phase [61]. Flexural strength, hardness, and resistance to brittle fractures are a function of the type, quantity, and morphology of the filler. Fillers also allow materials with high aesthetic value and good handling to be obtained [62,63]. Filler content in dental composites varies between 35–70% by volume and 50–85% by weight of the composite. The content by weight and volume, as well as the size, type, shape, and degree of dispersion of inorganic filler particles, vary between currently produced composites. Inorganic particles occur in fragments, plates, fibers, and in spherical form, as well as in the form of fine powder. The particle size of the filler varies widely from 0.007 to 70 microns, depending on the formulation [59]. In currently used dental composites, the inorganic phase consists of silica, quartz, borosilicate, lithium aluminum silicate, barium, aluminum, and strontium aluminum or aluminum oxides. Water in the oral environment may cause erosion of filler particles. While radiopaque glasses are known to dissolute in water and saline solutions, the most commonly-used fillers, silica and quartz, are comparatively inert in water [64,65].

The final properties of the composite dental material depend on the type of polymer matrix, the type and amount of filler, as well as the combination of the organic and inorganic phases. The interphase has a decisive influence on the physicochemical properties of the composite resulting indirectly from the proper homogenization of the filler particles in the polymer. Due to the highly hydrophilic nature of the surface of the filler particles, their dispersion in the hydrocarbon matrix is difficult. Surface modification of fillers prevents agglomeration, increasing the compatibility of the inorganic filler with dimethacrylate matrix [66,67].

To obtain optimal compatibility of inorganic filler, some physical and chemical surface modification methods have been suggested. Physical techniques, like ultraviolet laser and flame oxidation, are not so popular in dentistry. Therefore, most available products use chemical surface modification processes; this method employs chemical reactions between the hydroxyl groups existing on the particles and organic molecules, such as coupling agents or grafting polymeric chains [68]. The most popular coupling agents used in dentistry are silanes, which are characterized by the hydrolytically-active silicon-based functional group, RnSiXn (where n = 1 to 3). This unique class of organic silicon compounds can react with inorganic and organic substrates as well as with themselves and other silanes. This process is a complex hydrolysis-condensation reaction and leads to the formation of hybrid organic-inorganic structures [69]. It was found that hydrolytic degradation is drastically reduced with the addition of silane coupling agent at the appropriate concentration. The interface between fillers and the resin matrix is the fastest route for water diffusion into the interior layer of composite. Hydrolytic degradation occurs most readily in composite resin with no silane coupling agents. Although absorption of silanated fillers resists water diffusion [70,71], the absorbed water gradually hydrolyzes the interface silane and opens up an extra pathway for water diffusion [72].

**Figure 3:**
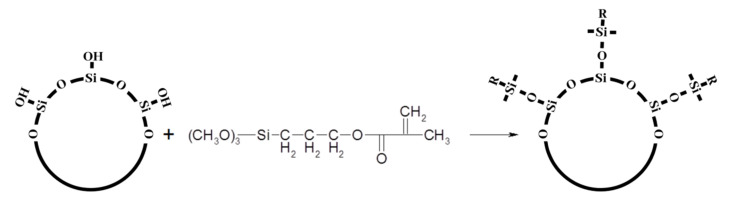
Silanization process of SiO_2_ with MPTMS silane. “R”—methacrylate group.

In methacrylic resin-based dental composites, the most popular silane coupling agent is 3-methacryloxypropyltrimethoxysilane (MPTMS). This substance reacts with the hydroxyl group on the filler surface and the methoxy group of other silane molecules (Figure 3). Additionally, MPTMS copolymerizes with polymer matrix by the methacryloxy functional groups [73]. An oxane bond (silicon-oxygen-silicon) is formed between the coupling agent and the inorganic filler (Figure 3). As it is a covalent bond with significant ionic character, it is far more vulnerable to hydrolysis than the bond between silane and the polymer matrix (carbon-carbon) [74]. MPTS is known as a sufficient coupling agent of the filler-matrix interface. However, as water diffuses into the composite material and there are additional substances in the oral cavity (acids, enzymes) which can accelerate the hydrolysis reaction, especially under cyclic loading, the hydrolytic stability of this coupling agent is a great concern [67].

During the degradation process of resin and coupling agent, changes in bulk microstructure are observed. Pores are formed via which monomers, degradation products, and additives are released. At the same time, the pH inside the pores is influenced by the acid–base functionality of the degradation products, thus accelerating the hydrolysis process [75]. Degradation of the interface results in fillers debonding, leaching of ingredients, micro cracks, increase in surface roughness, reduction of fatigue resistance, and final mechanical properties [76,77,78]. Therefore, the filler-matrix interface seems to be the weakest part of the composite material. Coupling agents with durable bonding and enhanced hydrolytic stability are still being sought.

## 6. In Vitro Ageing

Dental materials must withstand harsh conditions that also vary depending on the patient. Chewing habits, dietary factors, humidity, substances, and temperature fluctuations—all contribute to an unpredictable environment, which can affect the durability of materials. Hence, to predict behaviour of materials in the oral environment and the acceleration of ageing processes, tests which imitate the conditions prevailing in the oral cavity are needed. Table 1 presents the main directions of research on the ageing processes of composite materials. In most studies, material ageing was observed by increasing water sorption, leaching of degradation products and unreacted substrates, cracks, increased roughness, increased abrasiveness, colour change, reduced strength, and hardness (Table 1). These studies generally investigate usage of a selected ageing factor on certain mechanical, physical, or chemical properties of the material. The composition of the materials is the most important factor influencing the changes occurring in the material due to ageing in various environments.

The cellular and bacterial activity during ageing has also been investigated. In comparison to enamel and other restorative material (ceramics, metals), *streptococcus* mutants showed higher affinity for resin composites [79]. This microorganism was also found to demonstrate accelerated growth on resin composites in vitro [80]. This can be explained by surface roughness and the higher affinity of salivary proteins to polymeric materials. Additionally, some researchers claim that unreacted monomers and composite degradation products can promote the growth of several species of cariogenic bacteria [81,82]. *S. mutans* is capable of catalysing the hydrolysis of the resin matrix used in dental composites and adhesives; the bacteria increase esterase production in response to degradation products, thus accelerating the biodegradation processes [83]. The formation of a dense bacterial biofilm can also result in the ongoing destruction of resin composite because of the associated changes in surface roughness and degradation by the microorganism’s esterase activities [84].

The chemical environment can therefore have an appreciable influence on resin-based restoratives. The most commonly used solvents that influence the ageing of dental materials include water, artificial saliva, ethanol, and NaOH solution. Food and beverages also influence the properties of dental materials (Table 1). It was noted that other aqueous solvents may be more aggressive than water alone. Ethanol and NaOH solutions are indicated as chemicals accelerating the hydrolysis process.

Due to the complexity of the oral environment, more complex ageing protocols are needed. Unfortunately, only a small number of researchers combine selected environmental factors: most studies are known from the research of industrial composites. Accelerated artificial ageing protocols typically use long intervals of exposure to UV-B rays, humidity, and alterations in temperature [158,159,160]. Some studies try to use more accurate protocols to simulate ageing in mouth, such as protocols based on a pH-cycling regimen and tooth brushing cycles [161,162]. Colour stability was tested after combined effect of tooth brushing cycles, food-simulating solutions, and staining in coffee [163].

Static tests allow data to be obtained for a long period of time and they are simple to perform; unfortunately, this can lead to incorrect results. Dynamic tests better mimic clinical conditions and could be extremely valuable in predicting clinical performance. There are two currently-available systems that can reproduce dynamic changes of stresses, and these are used widely in international research; one is thermal cycling based on the standard ISO/TS 11405:2015, which defines the conditions of thermal ageing for testing of adhesion to tooth structure (number of cycles: 500 cycles; temperature: 5–55 °C, dwell time: ≥ 20 s). However, many researchers disagree with the guidelines included in the ISO standard, indicating that the temperature fluctuations are too extreme and that 500 cycles are not enough to represent adequate ageing time [164,165]. An attempt to standardize artificial ageing of dental cements using thermal cycling found the most efficient ageing procedure for tested material being thermal cycling (5 °C /55 °C /1 min) for four days, and that a storage in water for four days at 55 °C may be considered as a viable alternative to thermal cycling [166]. A second method uses fatigue tests, which requires more time than the standard strength test due to the application of cyclic loading. Variable cyclic load is an inseparable element of the oral environment due to the forces exerted by mastication, grinding, and shivering. A failure of restoration (or tooth) caused by single mechanical overload is possible, but more likely failure will happen due to repetitive loading of masticatory forces. Mainly, failures occur in conditions where load changes cyclically and usable ranges of strength value cannot be adequately predicted by measures of static tests. The most important aspect of fatigue tests is the fact that failures occur at stresses that are generally much lower than the strength defined by static tests [167]. For the average person, such stresses are repeated more than 3 × 10^5^ times per year. Therefore, resistance to fatigue failure should be taken into account during evaluation of restorative materials [77]. Unfortunately, there is a general belief that the fatigue strength of dental material can be calculated from the static strength; therefore, fatigue tests are not so popular in dentistry. This relationship is true, but mainly for metals, where the structure is very different to composites. Recent research suggests that the fatigue properties of resin composites may be very useful in predicting clinical performance [18,167].

In the literature, there is a very extensive collection of research dealing with the impact of the ageing process on the properties of dental materials. Unfortunately, there are no ageing procedures adopted that would be useful and effective for the estimation of material performance in clinical conditions.

## 7. Summary

The ageing process is an inherent element of the use of resin composites in dentistry. Unfortunately, despite the continuous improvement of materials, restorations still possess an insufficient lifespan. The following conclusions can be drawn from the literature review:

1. The hydrolysis of dimethacrylate resins proceeds relatively slowly in neutral pH; however, enzymes and acids accelerate this process. The most susceptible bonds for nucleophilic attack are ester bonds.
2. The bonds formed between a coupling agent and an inorganic filler are highly vulnerable to hydrolysis due to their significant ionic character. This process can be accelerated, especially under cyclic loading, by additional substances (acids, enzymes). Therefore, the hydrolytic stability of coupling agent remains a great concern among researchers.
3. Other aqueous ageing solvents (artificial saliva, ethanol, or NaOH solution) can be more aggressive than water alone. Given the very complex chemical composition of the oral environment, ageing tests that use water can only marginally help predict the clinical performance of biomaterials.
4. Systems able to reproduce dynamic changes of stresses (thermal cycling, fatigue tests) better mimic clinical conditions and could be extremely valuable in predicting dental composite clinical performance.
5. Determining ageing procedure and tests of dental materials is essential.

## Figures and Tables

**Table 1:** Relevant studies assessing the impact of different factors on dental composites.

Factor		Information and Main Observations	References
**Biological**	Enzyme	Chemical degradation of methacrylate polymers due to enzyme-catalyzed hydrolysis reaction of the ester bond.	[50,57,85,86,87,88]
	Bacteria	Mainly used: *Streptococcus mutans.* Increased surface roughness. Resin composite showed significantly higher bacterial adherence than human enamel. Leachability of degradation products.	[80,89,90,91]
**Chemical**	Artificial saliva	Increased surface hardness. Increased sorption value (in comparison to sorption evaluated in water). Filler leachability of composite specimens was higher after storage in artificial saliva than in distilled water. Decreased mechanical properties.	[92,93,94,95,96,97,98,99,100]
	Food and drinks	Colour changes. Reduction in surface hardness. Reduction in mechanical properties. Increased surface roughness.	[101,102,103,104,105,106,107,108,109,110,111,112,113]
	Water	Long-term ageing in water, slightly but significantly, reduced fracture toughness. Reduced hardness. Less long-term effect on reduction of elastic modulus and flexural strength.	[114,115,116,117,118,119,120,121,122]
	Ethanol solution	Alcohol can more easily penetrate the resin matrix. 75% ethanol is considered to be good solvent of dental composites. The Hoy’s solubility parameters of ethanol and the dimethacrylate resins, are similar (26.1 and 19.2–23.6 [J/cm^3^]^½^ appropriate) [123,124]. Elution of residual, unreacted monomers. Filler/matrix interfacial failure. Reduce mechanical properties.	[92,95,97,100,125,126,127,128,129,130,131,132]
	Sodium hydroxide	The solution of 0.1N NaOH (pH = 13) contains 1 million hydrogen ions more than distilled water or artificial saliva with pH = 7. Therefore, the degradation process occurs more intensively. Degradation of composite. Filler dissolution. Increase in wear. Decrease of composite strength properties. No imperceptible colour change.	[133,134,135,136,137]
**Physical**	Temperature	Thermocycling is a combination of hydrolytic and thermal degradation. Process negatively affected properties of resin-based dental materials (for example: flexural strength, fracture toughness, hardness, wear resistance).	[97,138,139,140,141,142,143,144]
**Mechanical**	Fatigue tests	Fatigue properties of resin composites may be very useful in predicting clinical performance. Linear elastic properties (elastic modulus, flexural strength, fracture toughness) are not good descriptors of the fatigue resistance of dental composite under cyclic bending. Fatigue properties are related to the type of filler, the silanization of the fillers, and the resin matrix. Fatigue fracture resistance of composites decreased after water and/or solvent immersion.	[77,145,146,147,148,149,150,151,152,153]
**Others**	Wet-storage arrangement	The wet-storage arrangement had a significant influence on mechanical and physical properties (biaxial flexural strength, hardness, water sorption, and solubility). Storage conditions should be considered as a variable in in vitro research of dental restorative materials.	[154]
	Strain rates during strength testing	Difficulty in comparing obtained results. May have an influence on mechanical properties.	[155,156,157]
